# Sustainability initiatives in inpatient psychiatry: tackling food waste

**DOI:** 10.3389/fpsyt.2024.1374788

**Published:** 2024-07-04

**Authors:** Timur Liwinski, Iona Bocek, Andreas Schmidt, Eva Kowalinski, Frieder Dechent, Franziska Rabenschlag, Julian Moeller, Jan Sarlon, Annette B. Brühl, André Nienaber, Undine E. Lang, Christian G. Huber

**Affiliations:** ^1^ University Psychiatric Clinics Basel, Clinic for Adults, University of Basel, Basel, Switzerland; ^2^ Division of Clinical Psychology and Epidemiology, Department of Psychology, University of Basel, Basel, Switzerland; ^3^ Central Institute of Mental Health, Medical Faculty Mannheim and University of Heidelberg, Mannheim, Germany

**Keywords:** food waste, hospital waste, health care sustainability, environmental sustainability, environmental stressors, water usage, water footprint, carbon footprint

## Abstract

**Background:**

Food plays a dual role in promoting human health and environmental sustainability. Yet, current food systems jeopardize both. Food waste poses a major global challenge due to its significant economic, social, and environmental impacts. Healthcare facilities generate the largest amounts of food waste compared to other forms of catering provision. Food waste correlates with environmental unsustainability and diminished patient satisfaction, compounding the prevalent challenge of hospital malnutrition and contributing to suboptimal patient outcomes.

**Materials and methods:**

In a three-year interventional study (2020-2022) at a psychiatric tertiary care center, we assessed and mitigated food waste using evidence-based measures. We conducted systematic food wastage audits over three years (2020-2022) in May and June, each lasting four weeks. Costs were analyzed comprehensively, covering food, staff, infrastructure, and disposal. Environmental impact was assessed using Umweltbelastungspunkte (UBP) and CO_2_e/kg emissions, alongside water usage (H_2_O - l/kg).

**Results:**

Economic losses due to food wastage were substantial, primarily from untouched plates and partially consumed dinners, prompting meal planning adjustments. Despite a >3% increase in meals served, both food waste mass and costs decreased by nearly 6%. Environmental impact indicators showed a reduction >20%. Vegetables, salad, and fruits constituted a significant portion of waste. Overproduction minimally contributed to waste, validating portion control efficacy.

**Conclusion:**

Our study highlights significant economic and environmental losses due to hospital food waste, emphasizing the importance of resource efficiency. The strategies outlined offer promising avenues for enhanced efficiency. The decrease in food waste observed over the three-year period underscores the potential for improvement.

## Highlights

- Our manuscript addresses a critical yet understudied aspect within psychiatric care—the impact of food wastage on patients in a large psychiatric tertiary care center. As part of the Swiss United Against Food Waste Initiative, our three-year study (2020-2022) delves into evidence-based interventions to assess and mitigate food wastage. Our findings reveal significant economic losses tied to untouched and partially consumed dinners, with implications for patient satisfaction and resource strain in psychiatric settings.
- Despite the unique challenges in psychiatric care, our interventions led to a nearly 6% reduction in both the mass of food waste and associated costs, even with a >3% increase in meals served. Environmental impact indicators demonstrated a noteworthy reduction exceeding 20%, aligning with the broader goals of sustainability in healthcare. Specifically, within the psychiatric context, our research sheds light on the challenges of food waste. The study emphasizes the importance of tailoring interventions to psychiatric settings, where considerations of patient preferences and therapeutic environments play a crucial role.

## 1 Background

Food can promote human health and contribute to environmental sustainability. Unfortunately, current food systems endanger both of these vital aspects (1). The United Nations Environment Programme (UNEP) defines ‘food waste’ as “food and its inedible parts removed from the human food supply chain” (2). Food waste is a prevalent issue across various societal domains and poses a substantial hurdle to the achievement of nutritional, economic, and sustainability objectives on a global scale (3). Food waste permeates the entire food supply chain, spanning from agricultural production and processing to distribution and consumption by the end user (4). Food waste within the food services industry has been labeled as an “unsustainability hotspot” (5). Food wastage represents a market failure, leading to the disposal of over US$1 trillion worth of food annually. Globally, one-third of food produced for human consumption is lost or wasted, totaling around 1.3 billion tons annually, valued at approximately US$1 trillion (6). It is estimated that food waste will increase by 33% by 2030, reaching 2.1 billion tons (7). Evidence shows that the 1/3 ratio of waste to production persists across both developed and underdeveloped countries, albeit with variations in the stages where losses are more pronounced. In developed nations, waste is more significant in the final stages of consumption, whereas in underdeveloped countries, it is prevalent during production and transportation (7). Throughout the EU27, food waste represents a growing concern, mounting to 89 million tons per year, corresponding to 180 kilograms (kg) per capita (8). Disposing of food waste in landfills generates significant amounts of methane and carbon dioxide, potent greenhouse gases that contribute to global warming (9). Food waste is therefore a serious environmental failure, contributing to an estimated 6-10% of global greenhouse gas emissions and utilizing nearly 30% of the world’s agricultural land (2, 10). The Swiss Federal Office for the Environment estimates that 25% of the environmental impact of the food supply system results from food waste (11). Food loss and waste constitute a significant component of agriculture’s impact on climate change, contributing to an annual release of 3.3 billion tons of carbon dioxide equivalent (CO_2_-eq) emissions (12). While food waste occurs on a large scale, up to 783 million people face hunger annually, and 150 million children under five suffer from stunted growth due to chronic malnutrition Thus, food waste also represents a lamentable human failure. Consequently, the prevention of food waste emerges as an utmost priority (13). The research concerning food waste holds paramount importance, evident in the increasing volume of reports and scholarly articles available online and within scientific journals (14).

Globally, nearly one billion metric tons of food waste were generated in 2019, with households contributing 570 million metric tons, comprising 61% of the total. Approximately a quarter originated from food services (including school and corporate canteens and hospitals), and 13% from retail (15). In the EU in 2021, an average of 131 kg of food per inhabitant was wasted, totaling 58.4 million tons of food waste, encompassing both edible and inedible parts. Household food waste constituted the largest share among all economic groups, accounting for 54% of the total food waste, equivalent to 70 kg per inhabitant. The remaining 46% was generated upstream in the food supply chain: 21% from food products and beverage manufacturing (28 kg), 9% from restaurants and food services (including hospital catering; 12 kg), 9% from primary production (11 kg), and 7% from retail (9 kg) (16).

Large facilities such as hospitals wield significant influence in the food system owing to their substantial purchasing power, resource consumption, and waste generation (17). Hospitals bear a duty to integrate human health and environmental concerns, yet historically, food waste within hospital settings has exceeded that of other sectors within the food supply chain (18). The proportion of food waste within the overall waste output of hospitals appears to have remained stable over time. Both older and more recent studies from diverse healthcare systems suggest that up to 50% of total hospital waste comprises food waste, encompassing both organic non-edible materials (e.g., vegetable peelings and bones) and edible food suitable for human consumption (e.g., leftover meals) generated daily by patients, healthcare workers, and visitors (19, 20). In 2013, healthcare was estimated to account for 9.8% of greenhouse gas emissions in the United States (21). Addressing food waste poses a significant challenge for hospitals, as they must simultaneously meet environmental goals such as reducing carbon emissions and minimizing food waste while operating within budgetary constraints, fulfilling dietary needs, and ensuring patient satisfaction (5, 17). Obstacles inherent in hospital food service models encompass limitations in accommodating specific therapeutic diets, coping with seasonal ingredient fluctuations, accurately predicting meal demand, adapting food service types, optimizing kitchen layouts, managing prolonged intervals between food procurement and consumption, and addressing issues related to over-provision or incorrect items. Additionally, the hospital environment influences food waste through service disruptions and factors such as patient conditions affecting food consumption (22–25). Plate waste, defined as the uneaten portion of meals left by consumers, attains concerning levels within hospital settings. A meta-analysis comprising 21 studies conducted in Indonesia revealed a median plate waste rate of 27.6%, with an upper range extending up to 88.7% (26).

Observations indicate that over half of the patients are deemed at risk for malnutrition, with nearly 40% already presenting signs of malnourishment (27). Malnutrition in hospitalized patients is a syndrome linked with significantly heightened risks of morbidity, disability, both short-term and long-term mortality, hindered recovery from illness, prolonged hospital stay and increased healthcare costs (28). Unconsumed food in hospitals poses significant risks of malnutrition among inpatients. Reduced oral food intake in hospitalized individuals notably contributes to the decline in nutritional status, which correlates strongly with various outcomes, including complications, length of hospitalization, survival rates, and healthcare expenses (29). A correlation exists between elevated levels of food wastage and reduced intake of energy and protein, consequently exacerbating malnutrition-related concerns within hospital environments (18, 30, 31). A recent review assessing the extent and consequences of hospital food waste in the Eastern Mediterranean Region (EMR), comprising six studies, revealed that the average food waste in the EMR accounts for 25.4% of the total food served. Alarmingly, plate waste from patients diminishes dietary intake, impacts nutritional status, and increases susceptibility to malnutrition (32).

The recent increase in attention to hospital food waste concerns has led to recent systematic reviews assessing food waste in hospital settings. A recent review, synthesizing 19 predominantly retrospective studies on hospital services to improve nutritional intake and reduce food waste, proposed that personalized meal services and efficient room service might enhance nutritional intake while reducing food waste. this underscores the potential for improvement in food supply and waste management within hospital settings, although further prospective and larger-scale studies are warranted (29). Carino et al. conducted a qualitative synthesis of 80 studies examining environmental sustainability throughout every stage of the hospital food supply chain. A significant portion of studies (n=27) explored strategies to enhance consumption and decrease food waste. Consistent findings revealed that comparing bulk trolleys to plated meal delivery resulted in reduced food waste. Several studies investigated room service, each finding statistically significant reductions in food waste. Additionally, both isothermal trolleys and Steamplicity were found to effectively reduce food waste (17). Nevertheless, there were no studies that assessed the environmental impacts of the foods procured and served in hospital settings. This is significant as food waste occurring later in the food supply chain exhibits greater carbon intensity compared to wastage at earlier stages, attributable to the energy and natural resources expended during transportation, processing, and preparation (33). Despite the plethora of studies on waste, the broader environmental ramifications of food waste do not seem to be the primary focus of published research. The systematic review conducted by Cook et al. on the types and characteristics of food-related waste management strategies implemented in hospital food service settings revealed four peer-reviewed and 81 grey literature sources (published outside traditional publishing and distribution channels) (34). The predominance of grey literature underscores a critical deficiency in rigorous peer-reviewed research addressing food waste mitigation in healthcare facilities. The authors deduce that while it is promising to observe some instances of hospitals adopting favorable waste management practices, these occurrences are relatively few. The review highlights that hospitals employ a varied array of strategies to handle their food and food-related waste. Results suggest that composting emerges as the most frequently utilized strategy in practice, despite ranking lower (less preferred) on the food recovery hierarchy (35).

Psychiatric hospitals are specialized facilities offering residential care for individuals with psychiatric disorders. Psychiatric hospitals continue to serve as a cornerstone of psychiatric care, particularly for individuals experiencing severe distress. Despite policy trends advocating for the reallocation of resources from standalone mental hospitals to community-based care, several countries, including France and Germany, expanded their provision of mental hospital beds from 2005 to 2011 (36). Indeed, numerous highly developed countries maintain relatively high rates of psychiatric hospital provision, such as Germany with 0.335, Norway with 0.577, Switzerland with 0.589, and Japan with as many as 8.314 psychiatric hospitals per 100,000 inhabitants (37). Despite their substantial role in the inpatient healthcare sector, to the best of our knowledge, none of the existing studies have investigated hospital food waste management within the framework of inpatient psychiatric care. Individuals with psychiatric disorders represent a vulnerable demographic concerning inadequate access to nutritionally adequate sustenance (38). Risk factors affecting psychiatric patients may include adverse effects of pharmacological treatments, sedentary lifestyle habits, inadequate dietary patterns, insufficient nutritional knowledge and skills as well as the inherent nature of the psychiatric illness itself, which varies according to the phases of the disease (39, 40). However, limited data exist regarding malnutrition in psychiatric populations, with the exception of studies focusing on eating disorders (41). Malnutrition was detected in 42.5% of patients admitted to an acute psychiatric unit (42). Given the critical position of psychiatric hospitals as central hubs of care for vulnerable patients, coupled with their significant impact on the inpatient healthcare landscape, effective management of food waste within these facilities emerges as a pivotal factor. This approach is essential for maintaining economic viability, promoting environmentally sustainable practices, and facilitating optimal patient nutrition to support the healing process. Achieving success in these areas requires a cohesive integration of strategies, ensuring that financial, ecological, and therapeutic considerations align harmoniously. However, as far as current knowledge extends, there has been no published investigation of the food waste burden within specialized mental health institutions, nor an evaluation of the effectiveness of strategies aimed at reducing food waste.

We aim to address this critical knowledge gap by undertaking the first-ever study in Switzerland to investigate the issue of food waste within a prominent psychiatric tertiary care center. Our primary objectives are as follows:

1. To quantitatively assess the total volume of food waste generated by our hospital’s meal delivery services.
2. To assess the effectiveness of measures targeting food waste reduction over a three-year period, commencing at its inception.
3. To discern whether specific Track Units (symptom- and syndrome-based, decentralized units) produce greater quantities of food waste than others.
4. To discern the most important sources of food waste in the context of psychiatric inpatient care.
5. To delineate the environmental consequences for society and the potential cost-saving opportunities for the hospital’s food service budget.

In pursuit of these objectives, we aim to pinpoint critical areas of concern and propose corrective measures to effectively address the issue of food waste within the domain of psychiatric hospital care. By focusing specifically on psychiatric care, our research aims to shed light on a population often overlooked in discussions of food waste within healthcare facilities. This study is primarily directed towards healthcare administrators, facility managers, and policymakers involved in the management and operation of healthcare facilities. Additionally, it may also be of interest to researchers, dietitians, and environmentalists concerned with improving resource efficiency and sustainability in healthcare settings.

## 2 Materials and methods

**Figure 1:**
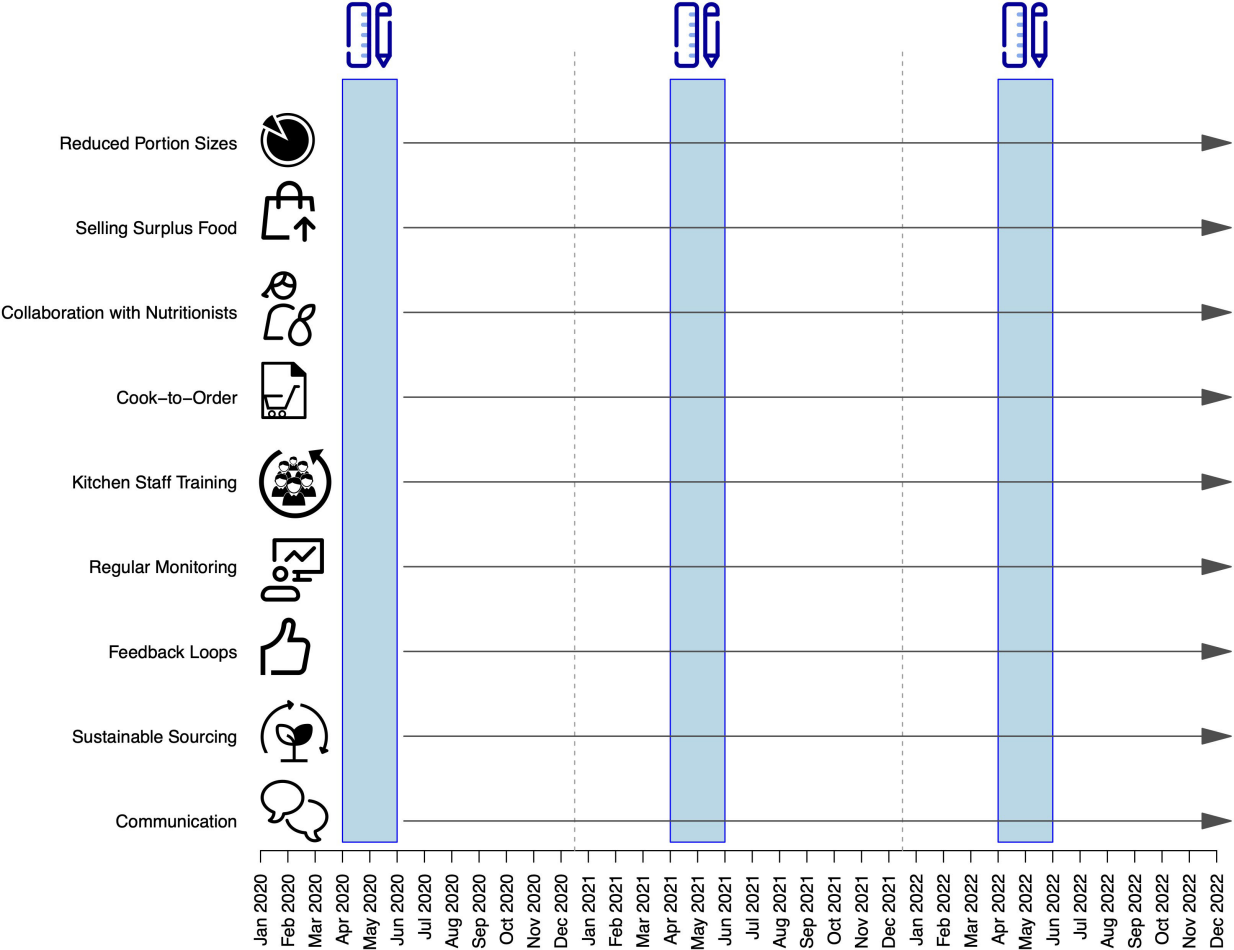
Study design. The blue rectangles represent the periods during which daily food waste measurements were taken. The x-axis indicates the timeline, while the y-axis highlights the interventions implemented to reduce food waste, initiated after the first measurement period.

We have adopted a prospective study design to collect data on food waste in our mental health center and have introduced evidence-based strategies to minimize food waste (**Figure 1**).

### 2.1 Study setting

The University Psychiatric Clinics (Universitäre Psychiatrische Kliniken, UPK) at the University of Basel, Switzerland, delivers psychiatric services to both inpatient and outpatient populations within the city of Basel and its neighboring regions, encompassing approximately 483,000 residents. Throughout the study period, the department maintained a capacity of 277 beds dedicated to inpatient care. Our center is organized in “Track Units”. These units are designed in a symptom- and syndrome-based, decentralized, and modular manner to cater to the patient’s unique stage-specific requirements for their treatment, spanning both inpatient and outpatient sectors (43). Our center provides food catering in various settings. Breakfast on the units is typically served as a buffet, prepared on-site with bulk deliveries. Lunch and dinner are delivered as individual plates. Additionally, in the staff restaurant, food is distributed through a service line, and there is also a salad buffet available.

### 2.2 Methodological background

On April 6, 2022, the Swiss Federal Council adopted an official action plan with the objective of halving food waste by 2030 compared to 2017. In pursuit of this goal, the federal government, on May 12, 2022, engaged in a cross-sectoral agreement with companies and organizations within the food industry, establishing specific reduction targets (11). Members of United Against Waste have been actively involved in shaping this agreement from its inception. This study adopted the methodological framework established by United Against Waste, an industry alliance within the Swiss food sector (44). It is dedicated to reducing food waste across the entire value chain.

### 2.3 Food waste quantification

To evaluate the impact of food waste and the effectiveness of measures to address it, we adopted the methodological framework established by the United Against Waste initiative, primarily developed by the Bern University of Applied Sciences (45). During a span of three years (2020, 2021, and 2022), we conducted food wastage measurements over four-week periods in the months of May and June. The results from these measurements were projected to reflect a full year (52 weeks), allowing us to observe trends over a three-year period. To ensure rigorous data collection, a content specialist from the Department of Environmental Protection offered survey input prior to conducting face-to-face interviews with nutrition managers from the selected hospitals. The audits involved the quantification of total food wastage in kg and the subsequent evaluation of associated economic losses in Swiss Francs (CHF). The analysis of the costs incurred by food waste includes a full-cost assessment, which means it considers not only the costs of food but also expenses related to personnel, infrastructure, and disposal. The price lists for food items were developed and consolidated by experts specializing in food waste consultation for gastronomy businesses (45). Raw data were collected using differentiated lists, subdivided for various categories of and individual food groups. The primary data source utilized was the Swiss Consumer Price Index for the corresponding months (46). We executed compositional analysis by sorting and categorizing food waste according to its respective food types. This methodology necessitated visual inspection and manual sorting. To quantify the food waste, we conducted physical weighing of the discarded items subsequent to segregating food waste from other waste streams, such as non-organic materials. This was followed by weighing the collected food waste using scales.

### 2.4 Quantification of the environmental impact

To gauge the environmental impact, we calculated the “Umweltbelastungspunkte” (UBP) per kg (47). The term “Umweltbelastungspunkte” denotes a recognized concept, often translated as “environmental load points” or occasionally as “ecological scarcity points”. UBPs constitute a methodology for capturing the life cycle assessment of a product. UBPs facilitate the representation of a product’s complex environmental impact in a single figure. They serve as a measure of the overall environmental burden, encompassing 26 types of emissions (e.g., CO_2_, methane, nitrous oxide, pesticides), the consumption of 8 resources (e.g., land, water), and the generation of hazardous waste. Each emitted substance is assigned an ecological weighting factor, where higher values denote a more hazardous environmental impact. Thus, UBPs provide a more comprehensive measure than the commonly used metrics of carbon dioxide emissions and water usage. In Swiss life cycle assessments, environmental load points serve as a reference metric (48). Furthermore, we computed the standard measure of carbon footprint, represented here as carbon dioxide equivalent emissions per kg (CO_2_e/kg). This measure signifies, for a given combination and quantity of greenhouse gases, the amount of CO_2_ needed to produce an equivalent global warming potential when assessed over a specified timeframe, typically 100 years. CO_2_ emissions are recognized as a crucial environmental indicator, as human CO_2_ emissions significantly contribute to global warming (49) and also affect the chemical composition of the world’s oceans (50). As an additional metric, we incorporated the water footprint, a concept originally introduced by Arjen Hoekstra, which assesses the extent of water use in relation to consumption by individuals (51). Here, we defined it as the total volume of freshwater use per kg of produced food waste (l/kg). As a reference for the water footprint of various food types, we relied on the data provided by the Water Footprint Network (52).

### 2.5 Evidence-based measures to reduce food waste

Starting at the commencement of the three-year assessment period, we introduced a range of research-backed strategies aimed at minimizing food waste (**Figure 1**). These measures include:

- Communication: We have actively communicated our food waste reduction efforts to staff, patients, and visitors, fostering awareness and garnering support (53).
- Sustainable Sourcing: Procurement of food from sustainable sources has been a key strategy, aligning with our environmental goals (54).
- Feedback Loops: Establishing a feedback system where patients and staff can provide input on food quality and preferences has allowed us to tailor our services and minimize waste (55). Feedback on food quality was included in the overall evaluation of the clinic stay, conducted at the end of the process. Patients rated the food quality on a ten-point scale, where 0 indicated “very poor” and 10 indicated “excellent.”
- Regular Monitoring: By regularly monitoring food waste and setting reduction targets, we have ensured ongoing improvements (54).
- Kitchen Staff Training: Training kitchen staff in food preparation techniques that maximize yield has been essential.
- Cook-to-Order: Implementing “cook-to-order” meal systems has significantly reduced overproduction.
- Collaboration with Nutritionists: The wards worked closely with nutritionists to develop appropriate meal plans, educate staff and patients about nutrition, and address any challenges related to food waste (56).
- Selling surplus food in the employee cafeteria at half price commences 30 minutes before the restaurant’s closing time.
- Reduced Portion Sizes: We have reduced portion sizes, with the option for patients with greater needs to order additional servings.

### 2.6 Statistical analysis

We used descriptive statistics to present counts and percentages, conducted correlation analysis using the Spearman rank test, and generated all statistical analyses and figures using the R statistical programming language (version 4.4.0).

## 3 Results

The analysis commences with an examination of the food waste burden, delineating its diverse sources and environmental repercussions at the conclusive assessment in 2022. Subsequent sections present a longitudinal evaluation aimed at evaluating the effectiveness of reduction measures implemented from 2020 to 2022, focusing on pivotal indicators.

### 3.1 Analysis of food waste and in-depth breakdown at the conclusive measurement

In the final evaluation conducted over a span of four weeks (May – June 2022), encompassing the general departments, the private clinic, and the personnel restaurant, the total number of meals provided reached 25,540. This led to the generation of food waste with a total mass of 4,089.49 kg, resulting in an economic loss totaling 98,147.76 CHF.

**Figure 2:**
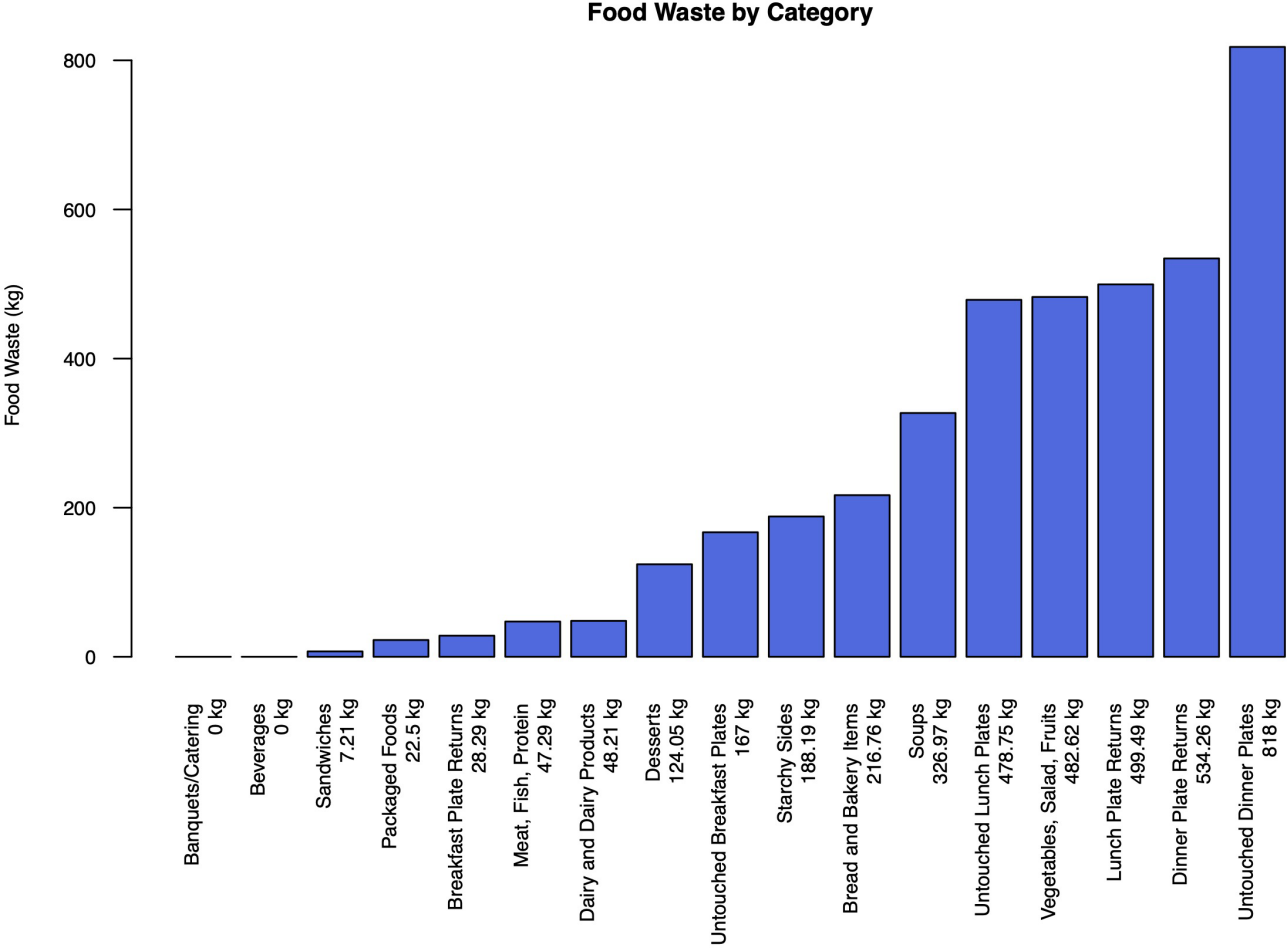
Food waste per category in kilograms during the last assessment period (2022), extrapolated for an entire year.

The category exerting the greatest influence on food waste was Untouched Dinner Plates, contributing 818 kg to the overall waste (**Figure 2**), representing a 20% share. Conversely, among diverse single food categories, Vegetables, Salad, and Fruits constituted the most substantial portion, accounting for 11.8% of the total waste.

Overproduction accounted for a relatively small proportion of food waste, contributing 5.64% to the total waste. Malfunctions such as spoiling or quality issues were negligible, with quality and equipment malfunction each contributing only 0.07% and 0.00%, respectively. The primary reason for food waste was Department Returns, which represented the lion’s share at 92.84% of the total waste.

In the breakdown of food waste sources, patients were the primary contributors, responsible for 3496.6kg (91.71%). Coworkers and visitors together accounted for 299.37kg (7.86%). Kitchen staff contributed 293.53 kg (7.71%).

### 3.2 Analyzing the ecological footprint of food waste

**Figure 3:**
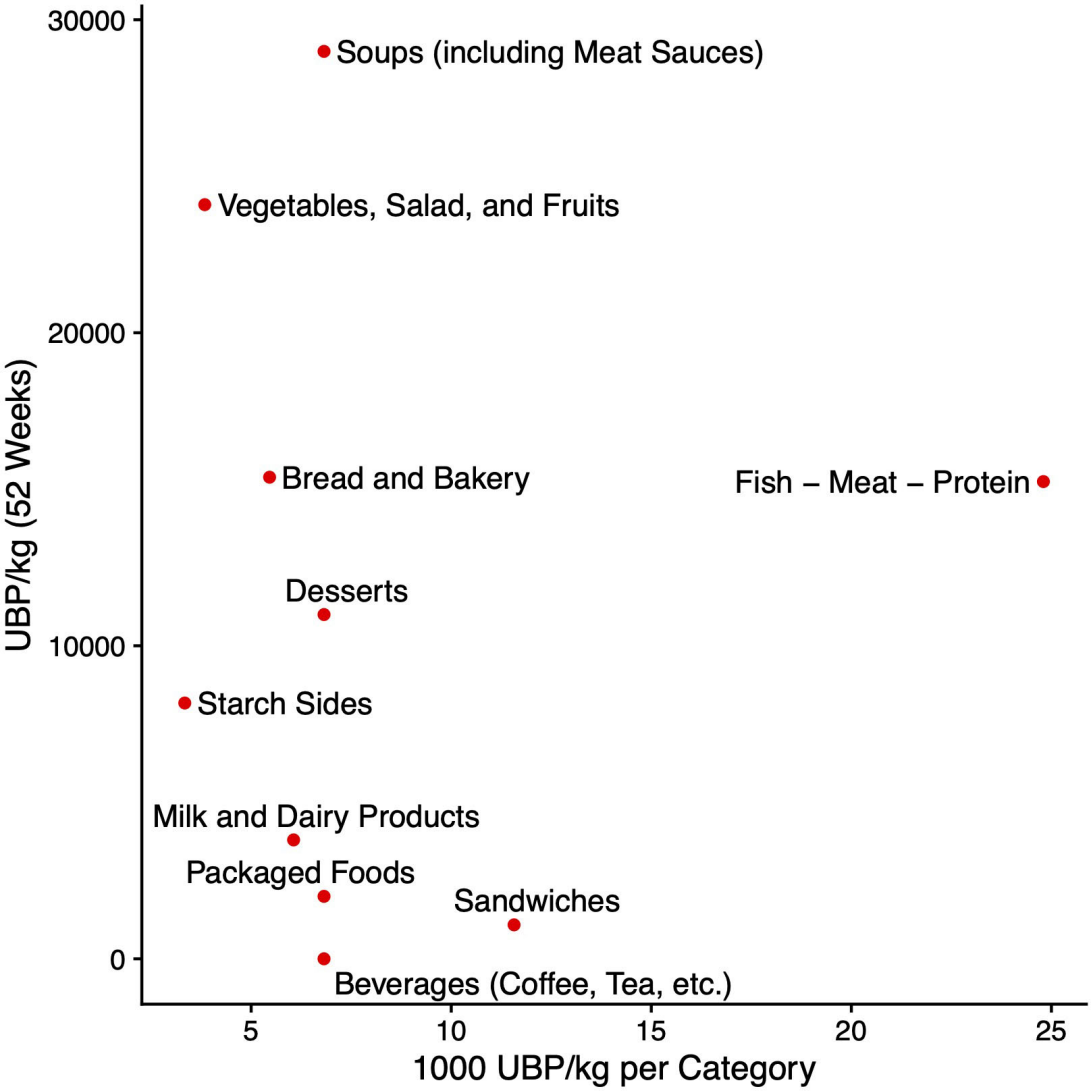
The x-axis displays the calculated Umwelbelastungspunkte (UBP) per unit (kg), while the y-axis shows the actual environmental burden in UBP at our center during the last assessment period, extrapolated for the entire year 2022.

There are significant variations in food waste quantities (measured in kg) and their corresponding environmental impact metrics across different food categories. Consistent with previous research, the “Fish, Meat, and Protein” category demonstrates the highest potential environmental impact per unit, with 24.80 thousand UBP/kg and 11.59 CO_2_e/kg. Conversely, the “Vegetables, Salad, and Fruits” category exhibits the highest water footprint per unit, at 170.00 liters per kg. However, no significant correlation was observed between impact per unit and total impact (r=−0.27, p=0.453). This is because serving sizes and the completeness of consumption by patients, as well as the amount of food discarded, must be considered (**Figure 3**).

**Figure 4:**
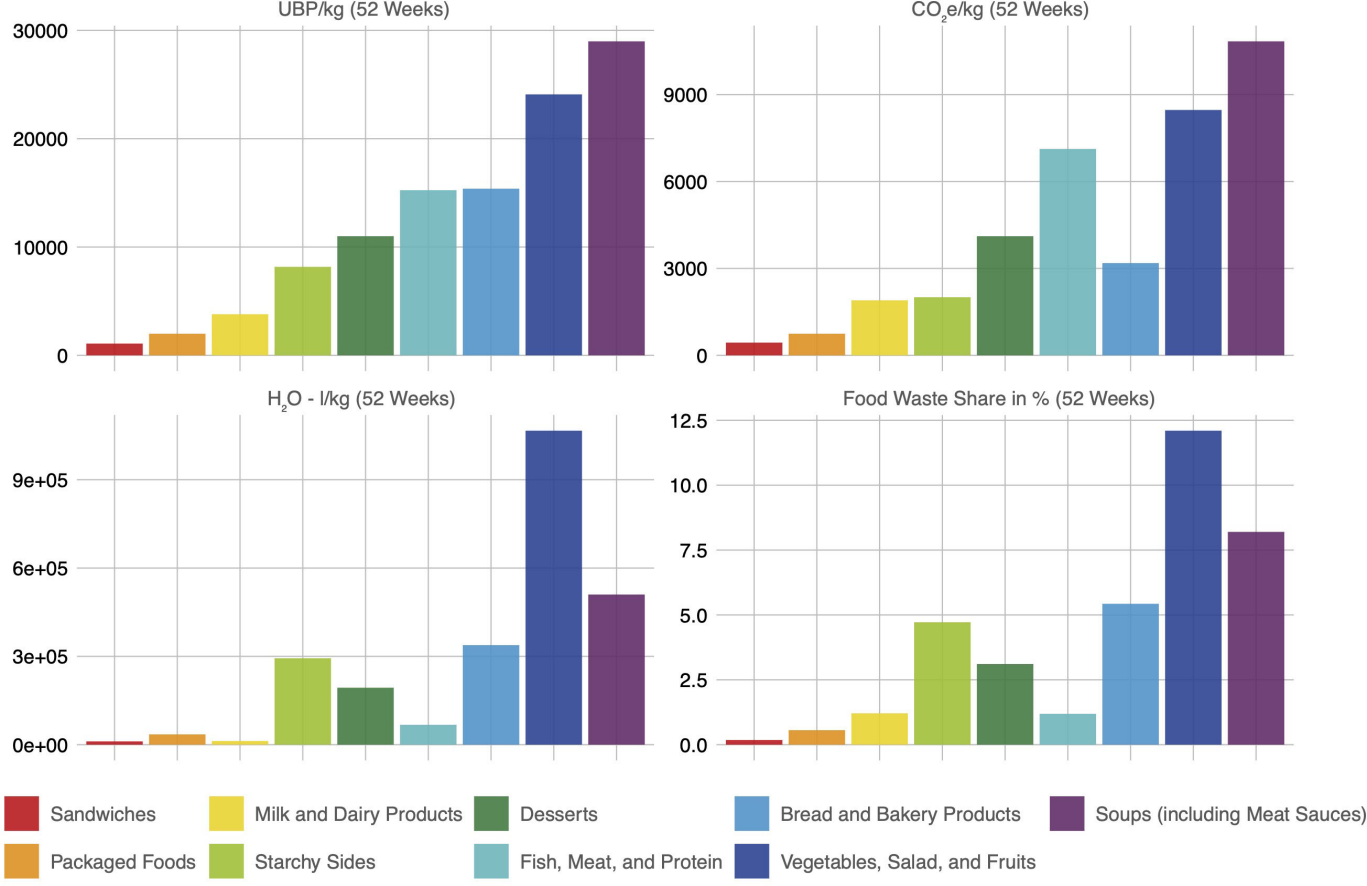
Environmental burden measured in Umweltbelastungspunkte (UBP), carbon footprint, and water footprint, along with the percentage share of mass by food category. Data was collected over a 4-week period (May to June 2022) and projected for 52 weeks (one year).

The “Vegetables, Salad, and Fruits” category exhibited the highest percentage share of total mass at 12.1%, followed by the “Soups and Sauces” category at 8.2%. Across all three environmental burden indicators, these two categories were the primary contributors to food waste burdens (**Figure 4**).

### 3.3 Comparison between measurement periods

**Figure 5:**
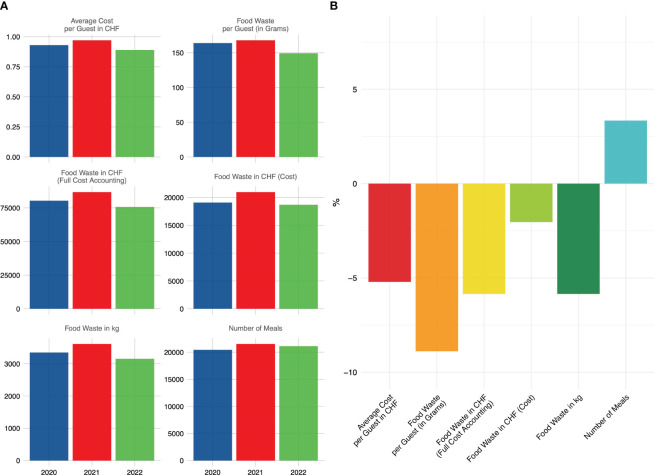
**(A)** Longitudinal assessment of food waste metrics, encompassing mass and cost, in conjunction with meal quantities, across the measurement periods. **(B)** Percental reduction in the food waste measures comparing the first and last assessment period. These data were collected during a four-week span in May and June of 2020, 2021, and 2022.

The longitudinal analysis was carried out, encompassing the general departments. Throughout the entire assessment period, we observed a decrease in the food waste impact, which suggests that the measures implemented to reduce it were effective: The total food waste in kg was reduced by -5.85%, the average food waste per guest by -8.89%. This remained true even though the total quantity of food served increased by 3.34% (**Figure 5**).

**Figure 6:**
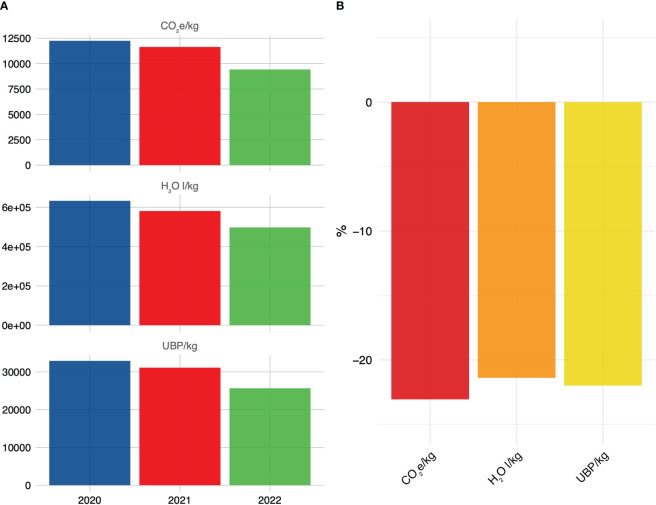
**(A)** Longitudinal evaluation of environmental burden related to food waste metrics over successive measurement intervals. **(B)** Percental reduction in the food waste measures comparing the first and last assessment period. These data were obtained over a four-week timeframe spanning May and June of 2020, 2021, and 2022.

In the context of environmental stressors, observations include a considerable decline in UBP per kg, dropping from 32,906.21 to 25,668.98, which amounts to a reduction of 21.99%. Similarly, the measurement for CO_2_e/kg declined significantly from 12,252.31 to 9,427.01, indicating a 23.06% reduction. Moreover, H_2_O (water usage) per kg decreased from 632,945.50 to 497,587.90, marking a 21.39% reduction. These results suggest a noteworthy improvement in environmental sustainability, with reduced resource consumption and emissions (**Figure 6**).

### 3.4 Longitudinal comparison between food items and track units

**Figure 7:**
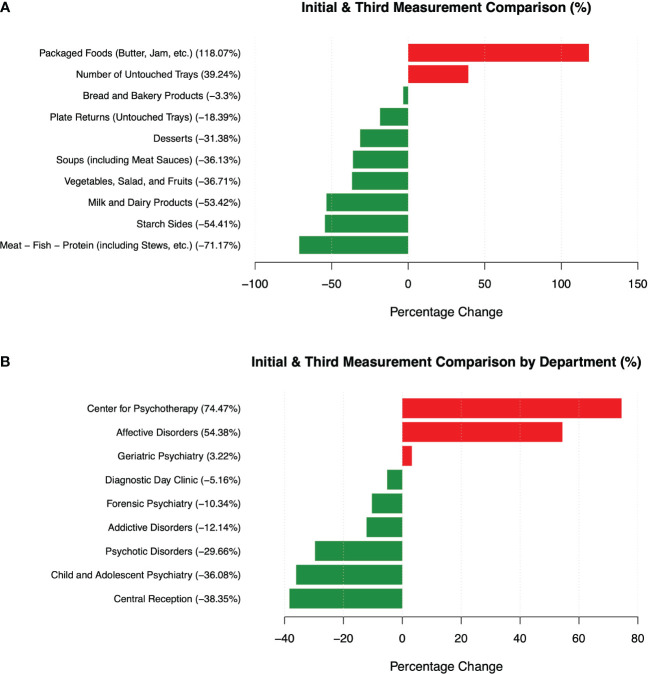
The percentage change in food waste mass is examined across two dimensions: **(A)** various food categories and **(B)** distinct departments.

In our longitudinal analysis of various food categories, notable improvements were observed in the reduction of food waste, particularly for Meat - Fish - Protein (including Stews, etc.) and Milk and Dairy Products, indicating effective waste reduction measures for these environmentally concerning food types. Conversely, a significant increase in waste was observed for packaged foods, accompanied by an overall rise in the number of returned untouched trays (**Figure 7A**).

During our examination of food waste patterns throughout the observation period, we observed that the highest initial burden of food waste was evident in the areas of forensic psychiatry, addictive disorders, and psychotic disorders. The most substantial reduction in food waste occurred in the central reception and the center for psychotic disorders, whereas the most significant increase was observed in the center for psychotherapy and the center for affective disorders (**Figure 7B**).

## 4 Discussion

Practically all discarded food in hospital settings is currently disposed of, with minimal composting or recycling efforts (57). This leads to the generation of costly organic waste that, when deposited in landfills, generates methane, a potent greenhouse gas (58, 59). Additionally, the surplus food wastage may negatively impact patient satisfaction, as patients often express distress over the significant volume of food being discarded (60, 61). As a result of these compelling factors, the reduction of food waste has become a top priority in hospital foodservice management (62). In our healthcare facility, the issue of food waste has emerged as a significant concern, impacting both economic and environmental aspects of our operations.

One of the salient findings of our research is the substantial economic loss associated with food waste in our hospital. The financial implications of such wastage are a critical consideration, particularly in healthcare settings where resources are often stretched thin (25). Among the noteworthy sources of food waste, untouched dinner plates and the return of partially consumed dinners have emerged as prominent concerns. The issue demands a detailed evaluation of dinner offerings and potential adjustments to meal planning and serving protocols. We propose three potential explanations for this finding. Firstly, certain patients may have engaged in dining activities with their families or friends as part of home-based training during the course of treatment. Secondly, dietary preferences, particularly a tendency towards refined carbohydrates, higher total fat alongside high carbohydrate consumption, and high sodium (commonly associated with fast food), as observed in individuals with psychotic and bipolar disorders (63, 64), could contribute to a higher proportion of meals being consumed outside the hospital setting, particularly during evenings when no daytime therapeutic activities are provided. Finally, the act of missing meals, which can be a symptom of an underlying condition such as depression, anxiety, or circadian misalignment, may also contribute to or worsen the trend we have observed (65). Our assessment reveals that vegetables, salad, and fruits constituted the largest proportion of food waste total mass and alongside soups and sauces had the highest negative environmental impact. High wastage also affects soups and sauces, which are mostly plant-based. This is a pivotal observation, as vegetables are universally acknowledged as fundamental constituents of healthful dietary patterns (66), and consequently, they play an indispensable role in nourishing patients and supporting their recovery (67). Recently, there has been an initiative to revamp hospital meals to simultaneously enhance nutrition for patients and staff, promote planetary health, and reduce costs (68). In line with recent policy changes and dietary guidelines (1, 69–71), there is a strong advocacy to reduce animal-based foods, particularly meat, and increase plant-based options in hospital menus (68, 72). Several states in the United States, including California and New York, have passed legislation requiring hospitals to offer plant-based options for patients (73, 74). Critics argue that reducing meat consumption and increasing plant-based food intake is crucial because livestock production accounts for approximately 14.5% of global greenhouse gas emissions (75). They also contend that the entire meat production process significantly contributes to breaching several planetary boundaries (76). Additionally, it is emphasized that meat production consumes large volumes of water and energy, accelerating the depletion of natural resources (77). Regarding dietary guidelines for healthcare menu planning, an analysis of guidelines from 85 countries concludes that most are not aligned with global environmental targets, such as the Paris Agreement (78). Research suggests that greenhouse gas emissions originating from animal-based foods are approximately double those emitted by plant-based foods globally. The production of food contributes approximately 17,318 ± 1,675 TgCO_2_e  yr^−1^ to global emissions, with 57% attributed to the production of animal-based food (including livestock feed), 29% to plant-based foods, and 14% to other uses (79). Yet, McAuliffe and team have raised concerns about conventional calculation techniques used to derive these figures (80). They advocate for an evaluation method that incorporates nutritional value and factors in the digestibility of essential amino acids. Employing this methodology, the environmental impact of certain animal-derived foods, like beef, has nearly halved. Conversely, the environmental footprint of plant-based products, such as wheat bread, has surged by nearly 60% (80). Food waste is often overlooked when debating the environmental impact of different food items. Nevertheless, it plays a substantial role, contributing to nearly 24% of greenhouse gas emissions and approximately 6% of total global greenhouse gas emissions (10). Wainaina and colleagues’ findings emphasize that the majority of food wastage primarily consists of fruits and vegetables (79%), followed by smaller proportions of meat and fish (8%), pasta and rice (5%), bread and bakery products (6%), and dairy items (2%) (81). As outlined by both the Food and Agriculture Organization (FAO) and existing literature, roughly 40% of fruits and vegetables experience loss between harvest and reaching retail markets. Additionally, post-retail stages witness a further 50% loss in this produce category (82–84). This underscores the importance of considering not just the environmental impact of production, but also consumer preferences and acceptance. There is minimal environmental advantage in producing a food item with reduced environmental costs if its eventual wastage contributes additional environmental burdens. Certain experts suggest that the key action for minimizing food waste in the healthcare sector is to prioritize plant-based options and decrease the consumption of animal products (85). Our data does not support this claim. Guinto et al., proponents of transitioning healthcare diets towards plant-based options, contend that certain developing nations may need to augment meat consumption due to the pervasive issue of protein energy malnutrition within those countries (72). We propose extending this approach to vulnerable subgroups within affluent countries as well. Despite being understudied and frequently overlooked in psychiatry, undernutrition and malnutrition remain prevalent issues. Data indicates that 32 – 48% of psychiatric inpatients are at risk of malnutrition (86, 87). Therefore, we suggest that it is essential to offer psychiatric inpatients appealing food that is both nutritious and well-balanced. Animal-sourced foods may play a crucial role in cognitive development and health (88). Meta-analyses indicate that vegetarian and vegan diets, as well as abstaining from meat, are associated with poorer mental health outcomes (89, 90). Prudently handled animal-sourced foods can be a valuable addition to nutritious hospital menus. Adesogan and colleagues argue that animal-sourced foods are essential. Sustainable development should address the needs of the poorest and most vulnerable populations while also balancing planetary health and sustainability goals (91). The reasons why fruits and vegetables are often discarded more frequently are unclear. Meat is highly preferred by most humans and generally viewed positively worldwide (92, 93). It has also been suggested that meat and fish are more palatable than plant-based staples (94). Despite this, the vitamins, minerals, fiber, and dietary bioactives in plant-based foods underscore their essential role in promoting overall health, including mental health (95, 96). In our center, the category of meat, fish, and protein saw the most substantial reduction in waste over the study period. In contrast, the reduction in plant-sourced food waste was only half as successful in percentage terms. To improve the acceptance of plant-based dishes, reduce waste, and encourage their consumption, hospital kitchens should focus on developing new, appealing plant-based meals and optimizing existing ones. At the same time, taste should be prioritized (85).

An encouraging aspect of our study is the relatively small contribution of overproduction to overall food waste. This applies to all single food item groups except for packaged foods, which have seen a concerning relative increase, although their overall contribution to food waste remains small. This achievement underscores the effectiveness of our strategies in meal planning and portion control. Our measures implemented to reduce food waste have yielded promising results, as indicated by our longitudinal assessment. Our results show a notable disparity in the efficacy of food waste reduction initiatives among different track units within our healthcare facility. Specifically, while Child Psychiatry and Psychotic Disorders have exhibited remarkable success in curbing food wastage, Affective Disorders have unfortunately experienced an upward trend in the percentage of discarded food. In seeking to elucidate the underlying reasons for these discrepancies and formulate strategies to narrow this variation, several key factors warrant consideration. Firstly, the variance in patient demographics and their distinct dietary requirements emerges as a salient factor contributing to the observed differences (97). Each track unit caters to a unique patient population, and this demographic heterogeneity significantly influences the generation of food waste (98). Notably, variations in patients’ clinical conditions and their associated eating habits emerge as a critical determinant (38). Clinical populations, particularly those with a history of disordered eating, may exhibit proclivities towards generating more food waste. Tailoring menus to align closely with patient preferences and specific dietary needs emerges as a potential solution, a paradigm notably exemplified by Affective Disorders. Furthermore, the implementation of tailored menu plans calls for stringent adherence to portion control and ongoing staff training. It is imperative that staff members, both in the kitchen and clinical departments, receive comprehensive training in the techniques of minimizing food waste. Regular refresher courses are integral in maintaining awareness and proficiency in these strategies. To continue our efforts in reducing food waste, we furthermore propose several future measures:

- Food Recovery: Partnering with local food banks or shelters to donate excess but safe-to-eat food can contribute to waste reduction (99, 100). Food waste in hospitals can be effectively repurposed through donations to individuals experiencing food insecurity (101, 102). Nonetheless, food hygiene regulations may impose restrictions on the reutilization of unserved food, and there is a shortage of literature addressing these specific practices (103).

- Food Recycling: Implementing a food recycling program to convert food scraps into compost or energy is an environmentally responsible option (104). Composting and anaerobic digestion offer the opportunity to repurpose food waste and contribute to the establishment of a circular food economy. Composting involves utilizing organic materials, such as food scraps, to generate a product that enhances soil quality and health (105).
- Menu Optimization: Regularly reviewing patient satisfaction and the menu to identify and remove unpopular items can further minimize waste (106).
- Patient Education: Educating our patients, especially in a vulnerable population, about the importance of minimizing food waste can be beneficial (107).
- Smart Kitchen Technology: Utilizing technology for precise ingredient measurements and portion control can enhance efficiency (108, 109).
- Furthermore, as part of our pilot program, we intend to experiment with providing food to patients in designated wards through the staff restaurant, ensuring prompt serving upon specific individual orders.
- We also recommend enhancing feedback loops by offering a more detailed questionnaire focused on various aspects of food quality and taste.

## 5 Limitations

Our study has several limitations. It was exclusively conducted within a single psychiatric tertiary care center, which restricts the applicability of our findings to a broader context. The absence of a control group or comparative analysis complicates the establishment of a direct causal link between the suggested corrective measures and the observed reduction in food waste. While the extrapolated longitudinal three-year measurement yielded valuable data, it may not encompass seasonal fluctuations or long-term food waste trends. To strengthen the evidence, additional studies are necessary to validate the enduring viability and practicability of the proposed corrective measures. This is imperative to guarantee their successful implementation and sustained effectiveness. Furthermore, it is important to highlight that during the years 2020 and 2021, the potential impact of COVID-19-related factors may have contributed to our results, rendering it challenging to draw definitive conclusions in this regard. Nevertheless, it is probable that in 2022, the influence of COVID-19 effects is not expected to be a significant factor. Research indicates that consumer-related food wastage decreased during the pandemic (110–112). As a result, it is possible that our initial assessment may have underestimated the food waste burden that would have existed under pre-pandemic circumstances. Nonetheless, it is worth noting that, to the best of our knowledge, there are no published food waste data from large healthcare facilities during the COVID-19 pandemic available for a dependable comparison. When addressing the limitations, it is important to consider that this analysis pertains to an affluent country, which may restrict the generalizability of the results to other (less affluent) countries and healthcare systems.

## 6 Conclusion

In conclusion, our research highlights the significance of addressing food waste in a healthcare setting. The economic and environmental implications of waste necessitate diligent and scientific management. The measures we have implemented have already shown promising results, and future initiatives promise to contribute to further waste reduction, ultimately benefiting our hospital, its patients, and the environment. By addressing food waste in psychiatry settings, healthcare providers can promote sustainable practices, enhance patient recovery and well-being, and optimize resource utilization.

## Data availability statement

The raw data supporting the conclusions of this article will be made available upon request to the corresponding author, without undue reservation.

## Author contributions

TL: Conceptualization, Formal analysis, Visualization, Writing – original draft, Writing – review & editing. IB: Conceptualization, Funding acquisition, Methodology, Supervision, Writing – review & editing. AS: Conceptualization, Methodology, Project administration, Writing – review & editing. EK: Writing – review & editing. FD: Writing – review & editing. FR: Writing – review & editing. JM: Writing – review & editing. JS: Writing – review & editing. AB: Writing – review & editing. AN: Writing – review & editing. UL: Writing – review & editing. CH: Conceptualization, Funding acquisition, Project administration, Resources, Supervision, Writing – review & editing.
